# Comparison of the Antioxidant Effects of Quercitrin and Isoquercitrin: Understanding the Role of the 6″-OH Group

**DOI:** 10.3390/molecules21091246

**Published:** 2016-09-19

**Authors:** Xican Li, Qian Jiang, Tingting Wang, Jingjing Liu, Dongfeng Chen

**Affiliations:** 1 School of Chinese Herbal Medicine, Guangzhou University of Chinese Medicine, Waihuan East Road No. 232, Guangzhou Higher Education Mega Center, Guangzhou 510006, China; Jiangqiande920711@163.com (Q.J.); wtttx0304@163.com (T.W.); 15622829151@163.com (J.L.); 2 School of Basic Medical Science, Guangzhou University of Chinese Medicine, Waihuan East Road No. 232, Guangzhou Higher Education Mega Center, Guangzhou 510006, China

**Keywords:** quercitrin, isoquercitrin, Q3G, 6″-OH, ω-OH, flavonoid glycoside, antioxidant mechanisms, mesenchymal stem cells

## Abstract

The role of the 6″-OH (ω-OH) group in the antioxidant activity of flavonoid glycosides has been largely overlooked. Herein, we selected quercitrin (quercetin-3-*O*-rhamnoside) and isoquercitrin (quercetin-3-*O*-glucoside) as model compounds to investigate the role of the 6″-OH group in several antioxidant pathways, including Fe^2+^-binding, hydrogen-donating (H-donating), and electron-transfer (ET). The results revealed that quercitrin and isoquercitrin both exhibited dose-dependent antioxidant activities. However, isoquercitrin showed higher levels of activity than quercitrin in the Fe^2+^-binding, ET-based ferric ion reducing antioxidant power, and multi-pathways-based superoxide anion-scavenging assays. In contrast, quercitrin exhibited greater activity than isoquercitrin in an H-donating-based 1,1-diphenyl-2-picrylhydrazyl radical-scavenging assay. Finally, in a 3-(4,5-dimethylthiazol-2-yl)-2,5-diphenyl assay based on an oxidatively damaged mesenchymal stem cell (MSC) model, isoquercitrin performed more effectively as a cytoprotector than quercitrin. Based on these results, we concluded that (1) quercitrin and isoquercitrin can both indirectly (i.e., Fe^2+^-chelating or Fe^2+^-binding) and directly participate in the scavenging of reactive oxygen species (ROS) to protect MSCs against ROS-induced oxidative damage; (2) the 6″-OH group in isoquercitrin enhanced its ET and Fe^2+^-chelating abilities and lowered its H-donating abilities via steric hindrance or H-bonding compared with quercitrin; and (3) isoquercitrin exhibited higher ROS scavenging activity than quercitrin, allowing it to improve protect MSCs against ROS-induced oxidative damage.

## 1. Introduction

**Figure 1:**
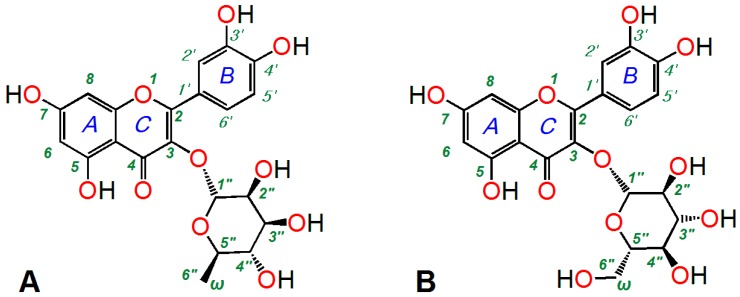
Structures of quercitrin (quercetin-3-*O*-rhamnoside, **A**) and isoquercitrin (quercetin-3-*O*-glucoside, Q3G, **B**).

Flavonoid glycosides can be found in a wide range of plants, especially those used in Chinese herbal medicines, and have been reported to exhibit remarkable antioxidant effects [1]. The glycosyl groups found in flavonoid glycosides are usually hexanoses, which are preferentially condensed in their six-membered (pyranosyl) ring forms. Among these pyranosyl rings, the glucopyranosyl and rhamnopyranosyl systems are regarded as two typical rings [2,3], because of their chemical stability. Flavonoid glucosides and flavonoid rhamnosides are therefore two of the most common members of the flavonoid glycoside family and can be found in a wide range of plants. For example, quercitrin (quercetin-3-*O*-rhamnoside, Figure 1A and Video S1) has been isolated from seven *Loranthaceae* plants [4], whereas isoquercitrin (quercetin-3-*O*-glucoside, Q3G, Figure 1B and Video S2) has been reported to be widely distributed in several *Moraceae* plants [5]. Furthermore, quercitrin and isoquercitrin have been reported to co-exist in several medicinal plants, including *Hypericum japonicum* [6], *Amomum* *villosum* [7], and *Polygonum hydropiper* [8].

Since stereo configurations of chiral carbons in the sugar chain do not actually influence the antioxidant effects [9], the difference between glucose and rhamnose can be reduced to the ω-OH group (more precisely, the ω-O atom) in rhamnopyranosyl. However, when a rhamnopyranosyl or glucopyranosyl unit is linked to a flavonoid moiety, the ω-position is numbered as C_6″_. For this reason, the ω-OH group in the rhamnopyranosyl compounds described in this study will be referred to as the 6″-OH group. As a ω-functional group in flavonoid glycosides, the 6″-OH has been largely neglected by researchers with an interest in the antioxidant activities of these compounds. In fact, most of the structure activity relationship studies pertaining to the antioxidant effects of these compounds have focused on the *A*, *B*, or *C* rings, with very few reports on the effects of the glycosyl moiety [10]. Furthermore, there have been no reports concerning the 6″-OH group of glycosyl units until now.

**Figure 2:**
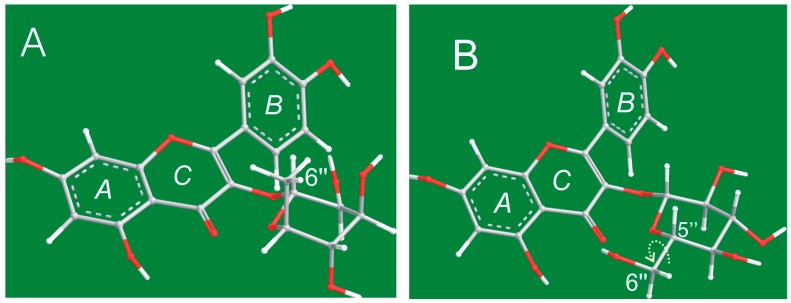
Ball-and-stick models of quercitrin (**A**) and isoquercitrin (**B**).

Interestingly, the ball-and-stick models suggested of quercitrin and isoquercitrin that the 6″-OH group may be a chemically active functional group. For example, the O atom of the 6″-OH group is highly electronegative (3.44) with two lone pairs of electrons. Furthermore, the 6″-OH group at the ω-position of the glycosyl ring apparently emerges out of the pyranosyl ring (Figure 2B), making it very different from the other three -OH groups (at the 2″-, 3″-, and 4″-positions) attached to the pyranosyl ring. Lastly, given that the σ bond between C_5″_ and C_6″_ can freely rotate (Figure 2B), the 6″-OH group could readily turn to its target site for reaction. With all of this in mind, we imagined that the 6″-OH group could play a number of versatile roles in the antioxidant activity of flavonoid glycosides.

To explore this possibility, we selected quercitrin and isoquercitrin as two model compounds to compare their antioxidant effects. We also used these compounds to conduct a mechanistic analysis of the role played by the 6″-OH group in antioxidant processes. Finally, we compared the cytoprotective effects of these two model compounds using mesenchymal stem cells (MSCs) to provide some biological evidence of the antioxidant effects of these compounds.

Given that MSCs have the potential to treat various diseases, especially those induced by reactive oxygen species (ROS) using stem cells transplantation engineering [11], it was envisaged that the results of this study would support the screening of flavonoid glycosides and their synthetic derivatives/analogues as effective antioxidants for cell transplantation engineering purposes. Furthermore, the results of this study could provide two candidates for the clinical application of MSCs in transplantation therapy, especially for ROS-induced diseases.

Finally, it is noteworthy that Mou and co-workers recently utilized a pyrogallol autoxidation assay to investigate the •O_2_^−^ radical-scavenging ability of quercetin at pH 8.2 [12]. However, the results of our previous studies have suggested that the pyrogallol autoxidation assay is strongly dependent on the pH at which it is conducted [13]. This dependence on the pH exists because samples bearing an acidic group such as a phenolic -OH group are sensitive to alkaline conditions. As illustrated in Figure 1A, quercitrin and isoquercitrin both contain several phenolic -OH groups that are weakly acidic. For this reason, any assays involving these compounds would be greatly distorted at pH 8.2, which could potentially result in erroneous experimental results [12]. In this study, we have conducted our experiments at physiological pH 7.4 (instead of pH 8.2) to improve the reliability of our results and provide a satisfactory explanation for the effects of quercitrin and isoquercitrin.

## 2. Results and Discussion

It has been well documented that ROS (especially •O_2_^−^ radical anions and •OH radicals) can be generated via Fe^2+^ catalysis according to the following equations (Equations (1) and (2)) [14]:

Fe^2+^ + O_2_ →Fe^2+^-O_2_ → Fe^3+^-•O_2_^−^ → Fe^3+^ + •O_2_^−^ (1)

Fe^2+^ + H_2_O_2_ → Fe^3+^+ •OH + OH^−^ (Fenton Reaction) (2)

**Figure 3:**
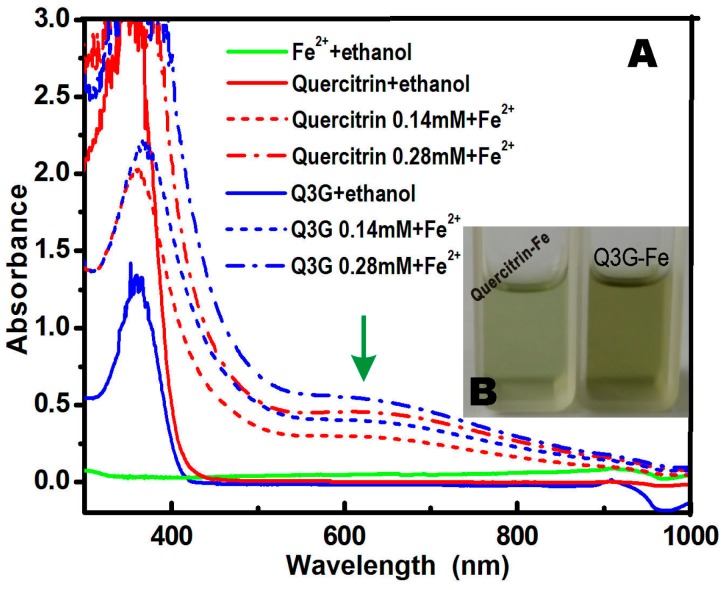
UV spectra of quercitrin and isoquercitrin (**A**); and the physical appearances of the quercitrin-Fe and isoquercitrin-Fe (Q3G-Fe) complexes (**B**).

Fe^2+^-binding (or chelating) can therefore efficiently reduce the generation of ROS and is considered by many to be an indirect approach to elicit the antioxidant activity of flavonoids and phytophenols [15]. In fact, Fe^2+^-chelating has been developed as a therapeutic approach for many diseases related to ROS [16]. In this study, quercitrin and isoquercitrin both bound Fe^2+^ ions efficiently to give strong UV absorption around 600 nm (Figure 3). This result indicated that quercitrin and isoquercitrin could undergo Fe^2+^-binding to inhibit the generation of •O_2_^−^. However, the UV spectra (Figure 3A) and physical appearances (Figure 3B) of the methanol solutions suggested that isoquercitrin had higher Fe^2+^-binding ability than quercitrin.

**Figure 4:**
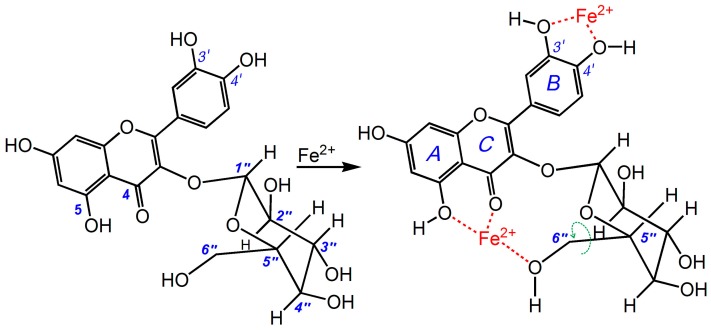
Proposed reaction of isoquercitrin with chelating Fe^2+^.

As shown in Figure 2, the 6″-CH_3_ group in quercitrin preferentially sat in an axial position (*a* bond), making it difficult for this group to access the flavone moiety (especially the *A* and *C* rings). It was therefore not possible for this group to participate in binding reactions at the 4- and 5-positions. However, the 6″-C and 6″-OH groups in the isoquercitrin molecule (Figure 2B) preferentially sat in an equatorial position (*e* bond). This orientation placed the 6″-OH group in close proximity to the flavone moiety (especially the *A* and *C* rings). Moreover, this conformation allowed for the 6″-OH group to swing to access the 4- and 5-positions via the free rotation of the σ bond between C_5″_ and C_6″_. The O atom of the 6″-OH could therefore participate in binding reactions at the 4- and 5-positions to form a stereo complex similar to that of EDTA (Figure 4). In fact, this reaction is formally characterized as a steric chelation reaction (not only a binding reaction). However, in the quercitrin molecule, there was only a planar Fe-binding interaction between the 4- and 5-positions with no similar steric chelation interaction. The difference helps to explain why isoquercitrin exhibited a much higher Fe^2+^-binding ability than quercitrin.

**Figure 5:**
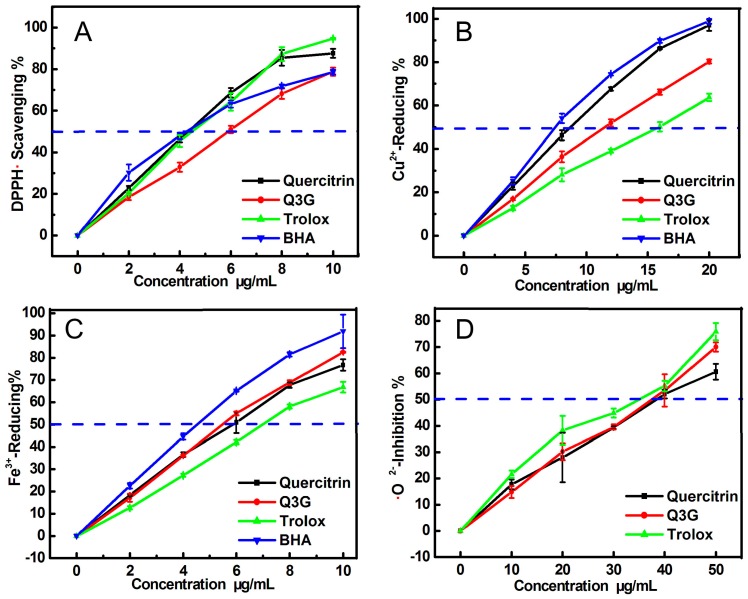
Dose-response curves of quercitrin and isoquercitrin (Q3G) in various antioxidant assays: (**A**) DPPH•-scavenging assay; (**B**) Cu^2+^ reducing power assay; (**C**) FRAP assay (Fe^3+^ reducing); (**D**) •O_2_^−^-scavenging assay. Each value is expressed as mean ± SD, *n* = 3. Trolox and BHA are used as the positive controls.

To verify whether quercitrin and isoquercitrin can directly scavenge ROS, we studied their radical-scavenging effects on DPPH• radicals, which do not require metal catalysis. The DPPH•- scavenging assay confirmed that quercitrin and isoquercitrin could efficiently eliminate DPPH• radicals (Figure 5A and Table 1). This result implied that quercitrin and isoquercitrin both exert antioxidant activities by undergoing direct radical-scavenging reactions. However, isoquercitrin was found to be less effective as a DPPH• scavenger than quercitrin. Previous studies in this area have suggested that DPPH-scavenging mainly involves hydrogen-donating (H-donating) pathways, leading to the formation of stable DPPH-H molecules [17]. However, in the isoquercitrin molecule, the 6″-OH group could combine with H• radicals to form H-bonding interactions that could hinder the H-donating ability of the phenolic -OH groups in the *A* and *B* rings, because the 6″-OH group has two lone pairs of electrons. Furthermore, steric hindrance from the 6″-OH group could have an adverse impact on the H-donating ability of the phenolic -OH groups.

It was recently reported that DPPH• scavenging could also include a minor approach, i.e., electron-transfer (ET) [18]. To explore the possibility of ET in the cases of quercitrin and isoquercitrin, we determined the Cu-reducing powers of these compounds. The presence of an ET pathway is critical to the conversion of Cu^2+^→Cu^+^, because the reduction of metal species in this way is well known to involve electron (***e***) transfer reactions. As seen in Figure 5B, both quercitrin and isoquercitrin gave good dose-response curves. This result therefore suggests that both of these compounds possess ET abilities, enabling them to scavenge ROS. However, recent results from the literature have also indicated that the ET approaches involved in Cu-reducing processes are generally accompanied by a proton (H^+^) transfer [19].

To determine whether quercitrin and isoquercitrin could undergo ET processes, we conducted a FRAP assay under acidic conditions at pH 3.6. The ionization and transfer of protons (H^+^) would be highly inhibited under these acidic conditions. The occurrence of a Fe-reducing reaction in an FRAP assay would therefore be considered as a minor ET process. The data in Figure 5C revealed that both quercitrin and isoquercitrin could reduce Fe^3+^ to Fe^2+^ in a concentration-dependent manner at concentrations in the range of 0.0–10.0 μg/mL. This result means that both of these compounds possess minor ET activity. However, the mere ET activity of isoquercitrin was higher than that of quercitrin, most likely because of the 6″-OH group in isoquercitrin. It has been hypothesized that the 6″-OH group in isoquercitrin could induce the flow of an “electron stream” through the flavone moiety, because the O atom of this group is highly electronegative (3.44) and can freely rotate on its σ bond. In contrast, quercitrin does not contain a 6″-O atom (or 6″-OH group), leading to its lower ET activity compared with isoquercitrin.

Although the effects of the 6″-OH group of isoquercitrin only appear to be negligible based on the analyses presented above, this group did lead to an increase in the •O_2_^−^-scavenging activity of isoquercitrin compared with quercitrin. According to the IC_50_ values, isoquercitrin (IC_50_ 78.16 ± 4.83 μM) exhibited stronger •O_2_^−^-scavenging activity than quercitrin (IC_50_ 87.99 ± 5.43 μM). This result therefore indicates that the inclusion of a 6″-OH group leads to an increase in the antioxidant activity of flavonoid glucosides. The total effects of having a 6″-OH group in these compounds can be attributed to the fact that their •O_2_^−^-scavenging activity would involve several antioxidant pathways, including Fe^2+^-binding [20], H-donating, ET [21], proton transfer [22], and even radical adduct formation (RAF) [23]. In addition, it is noteworthy that the IC_50_ value determined in the current study for quercitrin was significantly lower than that of Mou [12], who reported an IC_50_ value of 97.26 μg/mL (216.30 μM). This discrepancy further confirms the problems associated with pH interference during pyrogallol autoxidation.

The ratio values of IC_50,Trolox_:IC_50,Quercitrin_ and IC_50,Trolox_:IC_50,Isoquercitrin_ (Table 1) suggested both quercitrin and isoquercitrin possessed higher antioxidant ability than the positive control Trolox.

**Figure 6:**
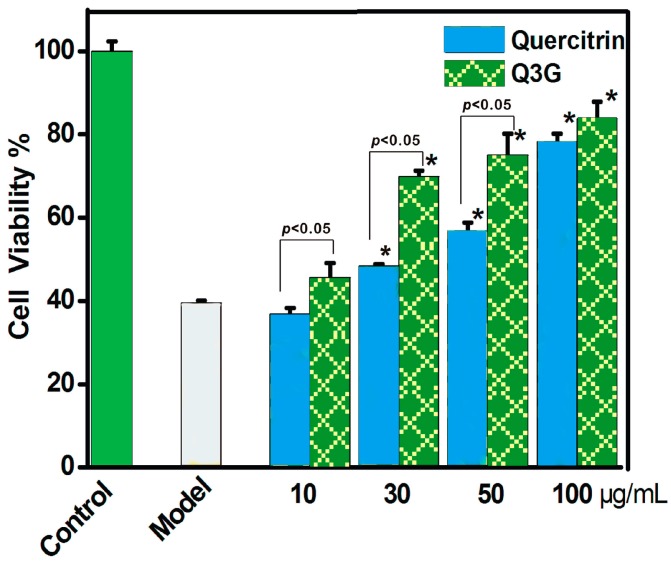
Protective effects of quercitrin and isoquercitrin against the Fenton reagent-induced damage of MSCs using an 3-(4,5-dimethylthiazol-2-yl)-2,5-diphenyl (MTT) assay. The Fenton reagent (FeCl_2_ plus H_2_O_2_) was used to generate •OH radicals. These data represent the mean ± SD (*n* = 3). * *p* < 0.05 vs. model.

Our assumption about the relative antioxidant levels of isoquercitrin and quercitrin was further supported by the 3-(4,5-dimethylthiazol-2-yl)-2,5-diphenyl (MTT) results obtained using an MSC-based model. In this assay, MSCs were initially oxidatively damaged using the Fenton reagent (FeCl_2_ plus H_2_O_2_), which was used to generate •OH radicals. The results revealed that quercitrin and isoquercitrin both protected the MSCs from •OH radical-induced damage at concentrations in the range of 0–100 μg/mL. However, isoquercitrin exhibited much stronger protective activity than quercitrin at the same concentrations (Figure 6).

The results of our MTT assay can also be used to explain our previous observations. For example, quercitrin can protect osteoblastic MC3T3-E1 cells against H_2_O_2_-induced dysfunction [24] and reduce UVB-induced cell death and apoptosis in HaCaT cells [25]. These results can be explained in the sense that H_2_O_2_ can lead to oxidative damage, whereas UVB irradiation can lead to the formation of large quantities of •OH radicals capable of exerting considerable toxicity. Furthermore, isoquercitrin can inhibit the H_2_O_2_-induced apoptosis of EA.hy926 cells [26]; several medicinal plants, including *Hypericum japonicum* and its injection (Tianjihuang Injection), exhibit hepatoprotective effects against CCl_4_-induced damage in rabbits [27].

## 3. Materials and Methods

### 3.1. Animals and Chemicals

Sprague-Dawley (SD) rats of four weeks of age were obtained from the Animal Centre at the Guangzhou University of Chinese Medicine, China. Quercitrin (C_21_H_20_O_11_, CAS number: 522-12-3, 98%) and isoquercitrin (Q3G, C_21_H_20_O_12_, CAS number: 482-35-9, 98%) were obtained from Sichuan Weikeqi Biological Technology Co., Ltd. (Chengdu, China). Pyrogallol, 2,4,6-tripyridyl triazine (TPTZ), (±)-6-hydroxyl-2,5,7,8-tetramethlychromane-2-carboxylic acid (Trolox), and butylated hydroxyanisole (BHA) were obtained from Sigma-Aldrich (Shanghai, China). MTT was from, Duchefa (Haarlem, The Netherlands). 1,1-Diphenyl-2-picrylhydrazyl radical (DPPH•) was obtained from Aladdin Chemical, Ltd. (Shanghai, China). 2,9-Dimethyl-1,10-phenanthroline hemihydrate (neocuproine) was obtained from J & K Scientific, Ltd. (Beijing, China). Tris-hydroxymethyl amino methane (Tris) was obtained from Dinggguo Biotechnology, Ltd. (Beijing, China). Dulbecco’s modified Eagle’s medium (DMEM) and fetal bovine serum (FBS) were purchased from Gibco (Grand Island, NY, USA). CD44 was purchased from Boster, Ltd. (Wuhan, China). H_2_O_2_, FeCl_2_·4H_2_O, CH_3_COONH_4_, FeCl_3_·6H_2_O, Na_2_EDTA, CuSO_4_, hydrochloric acid, and all of the other reagents were purchased as the analytical grade from Guangdong Guanghua Chemical Plants Co., Ltd. (Shantou, China).

### 3.2. Ultraviolet (UV) Spectra Determination of Fe^2+^-Binding

The Fe-binding effects of quercitrin and isoquercitrin were evaluated by UV spectroscopy. In these experiments, the Fe-binding reactions between quercitrin and isoquercitrin were monitored based on their UV spectra. Briefly, a 100–200 μL ethanolic solution of quercitrin (1 mg/mL) or isoquercitrin (1 mg/mL) was added to 1 mL of an aqueous solution of FeCl_2_·4H_2_O (5 mg/mL), and the total volume was adjusted to 1600 μL with 95% ethanol and mixed vigorously. The resulting mixture was then incubated at 37 °C for 10 min. The product mixtures were photographed using a camera (Olympus Pen, Shenzhen, China). The supernatant of each mixture was collected and analyzed on a UV/Vis spectrophotometer (Jinhua 754 PC, Shanghai, China).

### 3.3. DPPH• Radical-Scavenging Assay and Cu^2+^-Reducing Power Assay

The DPPH• radical-scavenging and Cu^2+^-reducing power assays were conducted according to previously reported procedures from the literature [28]. The experimental protocols, experimental apparatus, and formula for calculating the inhibition percentages were similar to those previously reported [28]. In contrast to this previous report, the samples being tested in this study were quercitrin and isoquercitrin, with Trolox and BHA being used as the positive controls. The final concentrations of quercitrin and isoquercitrin are shown in Figure 5A,B.

### 3.4. Ferric Ion Reducing Antioxidant Power (FRAP) Assay

The FRAP assay method used in this study was adapted from the method reported by Benzie and Strain [29]. This assay can be used to give an indication of the reducing ability of a material or mixture. The assay was performed in pH 3.6 buffer. Briefly, according to ratio of 1:1:10, the FRAP reagent was freshly prepared by mixing together 10 mM TPTZ and 20 mM FeCl_3_ in 0.25 M HOAc-NaOAc buffer (pH 3.6). The test sample (x = 10–50 μL, 0.1 mg/mL) was added to (100 − x) μL of 95% ethanol followed by 400 μL of FRAP reagent. The absorbance was read at 593 nm after 30 min of incubation at 37 °C against a blank consisting of acetate buffer. The relative reducing power of the sample compared with the maximum absorbance was calculated using the following formula.

(3) $$Relative\;reducing\;power\%=\;\frac{A-\;A_{min}}{A_{max}-\;A_{min}}\times 100\%$$

where, *A_max_* is the maximum absorbance, *A_min_* is the minimum absorbance, and *A* is the absorbance of sample.

### 3.5. Scavenging Ability towards •O_2_^−^ Radicals (Pyrogallol Autoxidation Assay)

The superoxide anion (•O_2_^−^)-scavenging activity was determined using a method previously developed in our laboratory [13]. Briefly, a 10–50 μL sample solution (1 mg/mL) was added to Tris-HCl buffer (0.05 M, pH 7.4) containing Na_2_EDTA (1 mM) and the total volume was adjusted to 990 μL using buffer. Ten microliters of pyrogallol solution (60 mM in 1 mM HCl) was added to the sample, and the resulting mixture was vigorously agitated before being analyzed at 325 nm every 30 s for 5 min. The •O_2_^−^ radical-scavenging ability was calculated as follows:

(4) $$Inhibition\%=\;\frac{\left(\frac{\Delta A_{325nm,control}}{T}\right)-\;\left(\frac{\Delta A_{325nm,sample}}{T}\right)}{\left(\frac{\Delta A_{325nm,control}}{T}\right)}\times 100\%$$

where *ΔA*_325*nm,control*_ is the increase in the *A*_325*nm*_ value of the mixture without the sample, *ΔA*_325*nm,sample*_ is the increase in the *A*_325*nm*_ value of the mixture with the sample and *T* is the time required for the determination (5 min in this case).

### 3.6. Protective Effect towards the ROS-Induced Damage of MSCs (MTT Assay)

The MSCs were cultured according to a previously reported method [28,30] with slight modifications. In brief, bone marrow was obtained from the femur and tibia of rat. The marrow samples were diluted with DMEM (LG: low glucose) containing 10% FBS. MSCs were prepared by gradient centrifugation at 900 *g* for 30 min on 1.073 g/mL Percoll. The prepared cells were detached by treatment with 0.25% trypsin and passaged into cultural flasks at 1 × 10^4^/cm^2^. MSCs at passage 3 were used for the investigation. The cultured MSCs were seeded into 96-well plates (4 × 10^3^ cells/well). After adherence for 24 h, the cells were divided into three groups, including control, model, and sample groups. The MSCs in the control group were incubated for 24 h in DMEM. The MSCs in the model group were injured for 5 min using FeCl_2_ (100 μM) followed by H_2_O_2_ (50 μM). The resulting mixture of FeCl_2_ and H_2_O_2_ was removed and the MSCs were incubated for 24 h in DMEM. The MSCs in the sample groups were injured and incubated for 24 h in DMEM in the presence of various concentrations of quercitrin and isoquercitrin. After being incubated, the cells were treated with 20 μL of MTT (5 mg/mL in PBS), and the resulting mixtures were incubated for 4 h. The culture medium was subsequently discarded and replaced with 150 μL of DMSO. The absorbance of each well was then measured at 490 nm using a Bio-Kinetics plate reader (PE-1420, Bio-Kinetics Corporation, Sioux Center, IA, USA). The serum medium was used for the control group and each sample test was repeated in five independent wells.

### 3.7. Statistical Analysis

The results were reported as the mean ± SD of three independent measurements, the IC_50_ values were calculated by linear regression analysis and independent-sample T tests were performed to compare the different groups. A *p* value of less than 0.05 was considered statistically significant. Statistical analyses were performed using the SPSS software 17.0 (SPSS Inc., Chicago, IL, USA) for windows. All of the linear regression analyses described in this paper were processed using version 6.0 of the Origin professional software.

## 4. Conclusions

Quercitrin and isoquercitrin can both behave as antioxidants in an indirect (i.e., Fe^2+^-chelating) and direct manner to scavenge ROS to protect MSCs against ROS-induced oxidative damage. In terms of the role played by the 6″-OH group in isoquercitrin, this group may lead to enhanced ET and Fe^2+^-chelating abilities compared with quercitrin, but lower H-donating ability via steric hindrance or H-bonding. Overall, isoquercitrin exhibited higher ROS-scavenging activity and greater cytoprotective effects towards MSCs than quercitrin. The present study may lead to the development of novel protectors in MSC transplantation engineering based on the structural modification of the 6″-position of flavonoid glycosides.

## Figures and Tables

**Table 1:** IC_50_ values of quercitrin and isoquercitrin in various antioxidant assays.

Assays	Quercitrin μg/mL (μM)	Isoquercitrin μg/mL (μM)	Positive Controls		Ratio (1)	Ratio (2)
			Trolox μg/mL (μM)	BHA μg/mL (μM)		
DPPH• scavenging	4.45 ± 0.17 (9.93 ± 0.38 ^a^)	5.89 ± 0.25 (12.68 ± 0.54 ^b^)	4.53 ± 0.11 (18.10 ± 0.44 ^c^)	4.42 ± 0.19 (24.53 ± 1.04 ^d^)	1.8	1.4
Cu^2+^-Reducing	8.91 ± 0.27 (19.87 ± 0.61 ^a^)	11.75 ± 0.36 (25.31 ± 0.78 ^b^)	15.68 ± 0.63 (62.66 ± 2.51 ^d^)	7.96 ± 0.28 (44.19 ± 0.69 ^c^)	3.2	2.5
FRAP	6.14 ± 0.29 (13.70 ± 0.65 ^b^)	5.71 ± 0.16 (12.30 ± 0.34 ^a^)	6.98 ± 0.11 (27.88 ± 0.47 ^d^)	4.61 ± 0.13 (25.60 ± 0.69 ^c^)	2.0	2.3
•O_2_^−^ scavenging	39.45 ± 2.43 (87.99 ± 5.43 ^b^)	36.30 ± 2.24 (78.16 ± 4.83 ^a^)	34.31 ± 0.90 (137.08 ± 3.61 ^c^)	N.D. N.D.	1.6	1.8

Each IC_50_ value was calculated by linear regression analysis of the dose response curves in Figure 5. The mass units of the IC_50_ values (μg/mL) were converted to molar units, and the resulting values are shown in parentheses. The linear regression was analyzed using version 6.0 of the Origin professional software (OriginLab Corporation, Northampton, MA, USA). Each experiment was performed in triplicate, and the IC_50_ values were presented as the mean ± SD (standard deviation, *n* = 3). Means values (μM) with different superscripts (a, b, c, d) in the same row were significantly different (*p* < 0.05). N.D., not detected. Ratio (1) = IC_50,Trolox_:IC_50,Quercitrin_; Ratio (2) = IC_50,Trolox_:IC_50,Isoquercitrin_.

## Supplementary Materials

Supplementary materials can be accessed at: http://www.mdpi.com/1420-3049/21/9/1246/s1.

## Abbreviations

The following abbreviations are used in this manuscript:

BHA: butylated hydroxyanisole
DMEM: Dulbecco’s modified Eagle’s medium
DMSO: dimethyl sulfoxide
DPPH: 1,1-diphenyl-2-picrylhydrazyl radical
EDTA: ethylene diamine tetraacetic acid
ET: electron transfer
FBS: fetal bovine serum
FRAP: Ferric ion reducing power assay
MSCs: mesenchymal stem cells
MTT: [3-(4, 5-dimethylthiazol-2-yl)-2,5-diphenyl]
Q3G: quercetin-3-*O*-glucoside
RAF: radical adduct formation
ROS: reactive oxygen species
SD: standard deviation
SPSS: statistical product and service solutions
TPTZ: 2,4,6-tripyridyl triazine
Tris: tris-hydroxymethyl amino methane
Trolox: (±)-6-hydroxyl-2,5,7,8-tetramethlychroman-2-carboxylic acid
